# DM-FS: A Comprehensive Database on Death-Modulated Fatal Shootings

**DOI:** 10.1038/s41597-025-04649-x

**Published:** 2025-03-03

**Authors:** Jacob Verrey

**Affiliations:** Cantab. Institute of Criminology, University of Cambridge, Sidgwick Ave, Cambridge, CB3 9DA UK

**Keywords:** Society, Social sciences, Scientific community, Human behaviour, Interdisciplinary studies

## Abstract

In the USA, officers and civilians engage in fatal encounters, situations in which a member of one group is slain at the hands of the other. Research on fatal encounters spans disciplines and can save lives, yet it tends to draw from databases that may be partial, unidirectional, and contain methodological differences. To remedy these issues, we propose a database on Death-Modulated Fatal Shootings (DM-FS). DM-FS makes five substantial contributions, and we highlight three. First, DM-FS is bidirectional. It is one of the few databases that enable the empirical exploration of how death in one group, police, affects deaths in another, fatally shot civilians and vice-versa. Second, DM-FS is comprehensive. It contains the entire universe of applicable agencies between January 1, 2015, and December 31, 2020. Third, it may be less partial than the status quo. Its rigorous validation procedures detected and corrected discrepancies between existing databases of fatally shot civilians: Fatal Encounters, Mapping Police Violence, Washington Post. DM-FS use cases are explored, such as in policymaking, modeling, and psychology.

## Background & Summary

In the United States, civilians and law enforcement engage in fatal encounters–encounters in which a member of one group is slain at the hands of the other. US officers, for example, fatally shot 4,915 civilians between 2015 and 2019^1^, whereas US civilians have slain 256 officers during the same period^2–6^. Research on these fatal encounters is highly consequential because it can save lives^7^, and it has attracted interest and analyses that span the social sciences and broader scientific community. Indeed, its research has invoked statistical modeling to discover risk factors^8–10^, psycho-mechanistic approaches to understand biases^11–13^, and even government, legal, and societal-level analyses to elucidate structural factors that could serve as the basis for an intervention^7,14,15^. Unfortunately, a key bottleneck hinders the development of this consequential research: the issue of data.

In other words, a handful of governmental and/or private databases tend to serve as the foundation for extant research on fatal encounters^16^, with many studies drawing exclusively from one database alone (e.g.,^17–21^). Unfortunately, these databases suffer from two major limitations that could distort research findings: (i) they may be partial and (ii) they take a unidirectional approach. First, there is a lack of impartiality in fatal encounters databases. Indeed, numerous databases exist^1,22–26^, yet many of these databases suffer from well-documented underreporting issues^16,27^; others are hosted by organizations that may have a political agenda (e.g.^28–30^); and there are methodological differences between databases concerning what gets reported (see methods). The political, methodological, and underreporting biases of these databases may influence research findings, possibly confounding results in certain cases that draw exclusively from one database.

Second, extant fatal encounter databases tend to be unidirectional; they tend to only record one aspect of the phenomena, such as exclusively recording civilians slain by officers^1,22,25^ or officers slain by civilians^23,24,26^, but seldom both. This is problematic because deaths in one group may modulate deaths in the other. The death of a civilian, for example, could motivate civilians to kill officers to exact revenge–a reality that has materialized across major US cities in recent years (e.g.,^31–33^). The death of an officer, on the other hand, may likewise impel officers into killing more civilians out of a mechanism like fear–as was the case with an ex-Dallas officer, who fatally shot an unarmed man^34^ not too long after five of her officer-colleagues were slain by a civilian^3,33^. Regardless of the mechanism, research cannot explore how death in one group affects deaths in another if extant databases typically only focus on one group. A bidirectional database, one that takes into account deaths on both sides, would remove this bottleneck.

To remedy the partiality and unidirectionality of existing databases, we propose a database on Death-Modulated Fatal Shooting (DM-FS). DM-FS allows researchers to explore how deaths in one group – officers – affect deaths in another – civilians – and vice-versa. Namely, DM-FS is broken into two related databases:

- **DM-FS Officers** enables the exploration of how an officer’s death affects the number of civilians other officers fatally shoot each year, and under which circumstances. For example, DM-FS Officers can be used to explore whether a civilian slaying an officer predicts that officers in his/her agency will engage in more fatal shootings in the subsequent year, potentially providing an empirical basis for the Dallas story^34^.
- **DM-FS Civilians** enables the exploration of how a civilian’s death affects the number of officers that other civilians kill each year, and under which circumstances. For example, DM-FS Civilians can be used to explore whether an officer fatally shooting a civilian impels local civilians to go out and fatally shoot an officer from the perpetrating agency, potentially providing an empirical basis for news stories depicting the civilian-initiated killings of officers^31–33^.

Given that civilians kill US officers each year^2–6^ and US officers fatally shoot civilians each year^1^, our database lays the foundation by which the relationship between the two ongoing phenomena may better be understood.

More concretely, DM-FS makes five substantial improvements over existing databases. First, DM-FS is one of the few fatal shooting databases that is bidirectional. Namely, it allows one to explore the interdependence between two phenomena –officer deaths and civilian deaths - that are typically examined independently. Second, DM-FS is comprehensive. It contains the entire universe of death-modulated fatal shootings from January 1, 2015, until December 31, 2020, allowing researchers to scrutinize the totality of the phenomenon. Third, DM-FS is uniform. It *standardizes* civilian deaths so that they are *relative* to an officer’s death date and vice-versa, enabling the comparison between events that otherwise experienced a disparate death date. Fourth, DM-FS is extensive. DM-FS carefully draws from other reports so that researchers can isolate potential explanations behind why fatal shootings change in response to death. Finally, and perhaps most importantly, DM-FS is less partial than the status quo. Indeed, we wrestled with multiple databases on slain officers and civilians and chose the one that best measured reality; and, when that could not be determined, we used multiple databases as a means of correcting for the methodological and reporting biases inherent within each. There may never be a truly impartial fatal shooting database, yet DM-FS may make a major step in that direction.

**Fig. 1:**
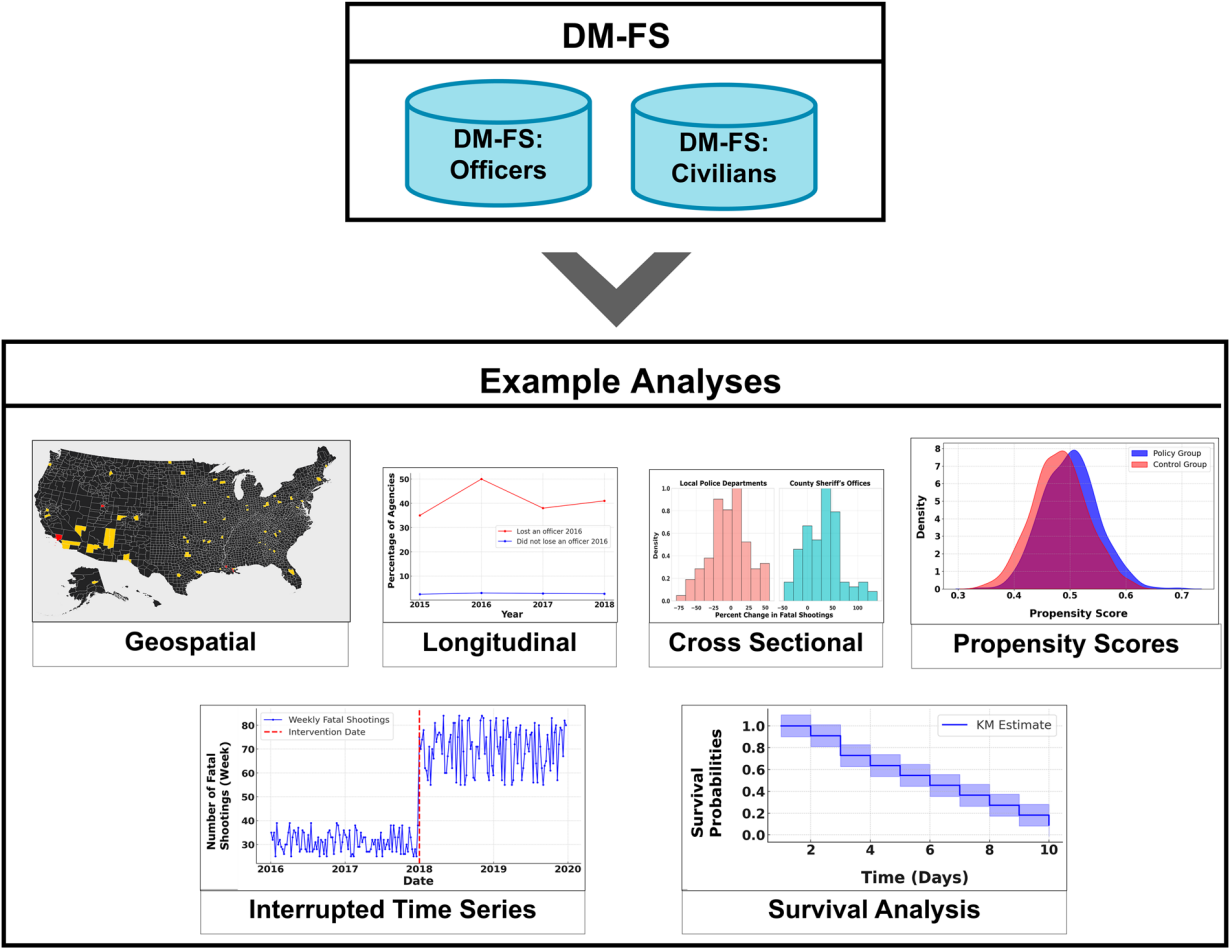
Illustration of Analyses that Can Be Conducted or Facilitated via DM-FS. Blue cylinders indicate a database that we created, whereas white squares indicate grouping. The flow suggested by the grey arrow indicates analyzing DM-FS Officers and DM-FS Civilians under the example analyses.

For these reasons and others, we envision that DM-FS can be used by both fatal shooting scholars and others throughout the social science community. To provide a few stylized examples, it can be used by policymakers and think tanks, who can use this database to devise tools that flag communities as at risk of an increase in fatal shootings; by psychologists interested in understanding mechanisms behind how death in one group modulates death in another, such as fear or revenge^35,36^; and even by aspiring authors and investigative journalists, as the idea of one death spawning another has inspired well-disseminated literary works like *Hamlet* and *Beowulf* as well as news stories^35^–it can provide an empirical basis of their next piece. To investigate these topics, those in the social science community can undertake a range of analytical techniques with DM-FS–techniques ranging from geospatial analysis to even an interrupted time series design, with further analytical examples appearing in Fig. 1.

## Methods

### Ethics

The Ethics Committee of the Institute of Criminology, University of Cambridge approved all aspects of this research, including all protocols, guidelines, and regulations followed, as well as methods used to conduct the data collection, and they also provided ethical oversight. As such, all methods were performed in accordance with these relevant guidelines, regulations, and protocols. Moreover, our database derived its contents from legacy data that was collected and reported on as part of the standard democratic, journalistic reporting process. Due to the retrospective nature of the database, the infeasibility of other research designs, and the minimal risk of harm, the need for informed consent was waived by the Ethics Committee of the Institute of Criminology, University of Cambridge. Finally, ethics approval was obtained via the standard application process: by submitting the full ethics application to the Institute of Criminology, University of Cambridge (B/Ethics/February Version 9), along with a completed risk assessment (RA7.12) and research abstract, before being reviewed and ultimately approved by the committee.

To further discuss ethics, all data used to construct DM-FS was extracted from the public domain. Indeed, DM-FS mainly drew from secondary databases, which, in turn, extracted information from various news stories, court cases, and government reports. Much of the content of DM-FS is a derivative of that work. For example, rather than listing the narratives of slain officers^2–6^, DM-FS transforms this data into a tabular format, thereby representing a derivative of the original source.

### Overview

**Fig. 2:**
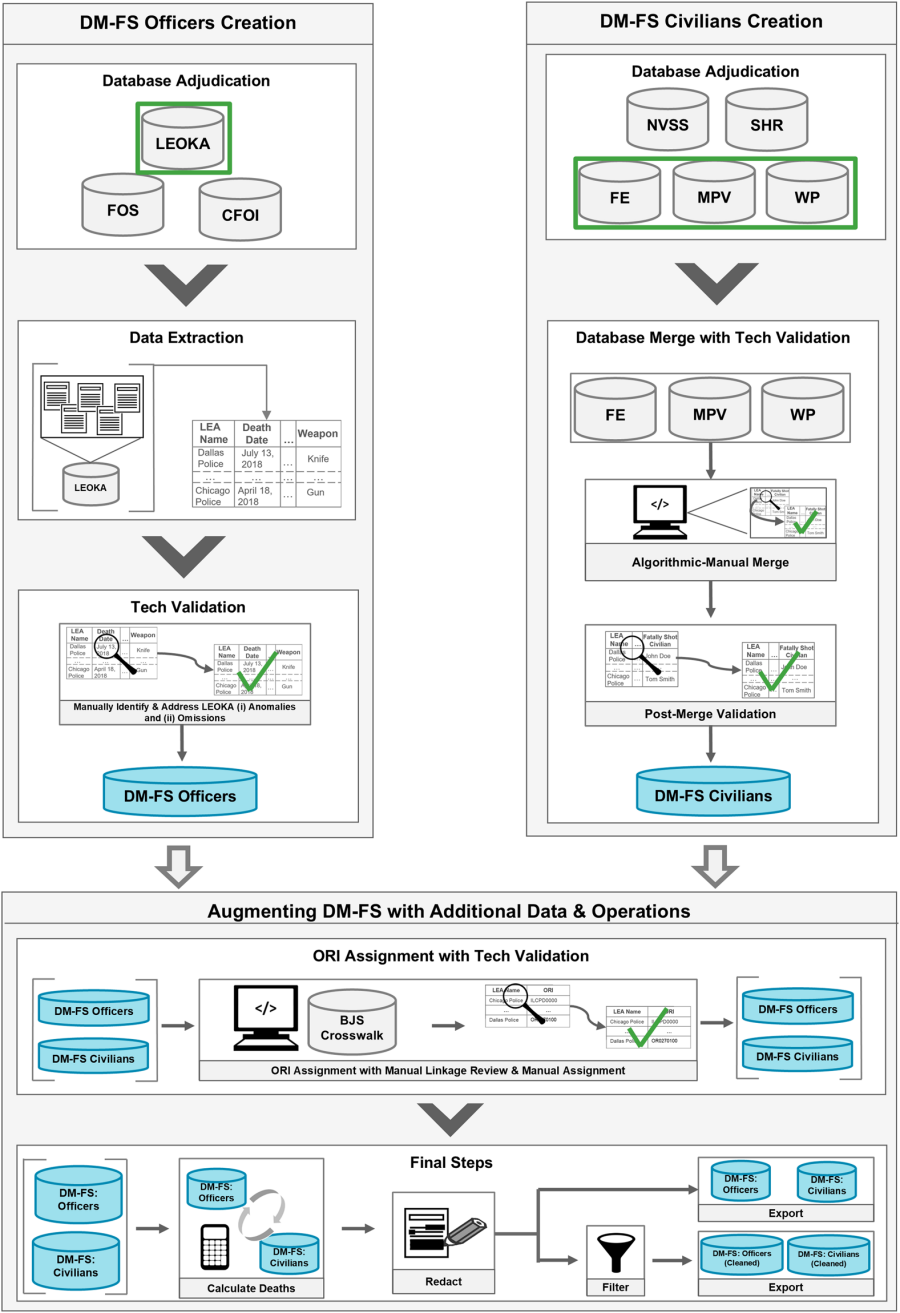
Schematic Overview of Database Construction. DM-FS was constructed via three broad sections, illustrated by grey boxes. Each section has various subsections, illustrated by white boxes. Cylinders represent databases: gray cylinders indicate the database was taken online, whereas blue indicates the database was created in-house. A green rectangle indicates that a particular database or databases was/were selected for use in the final database’s construction. Arrows indicate advancing from one section/subsection to another.

An overview of the construction of DM-FS appears in Fig. 2, with a list of acronyms appearing in Table 1. To construct DM-FS, we first constructed and technically validated both DM-FS Officers and DM-FS Civilians independent of each other. After passing the technical validations, we augmented both databases with additional information and performed another technical validation exercise to validate the efficacy of this augmentation. Next, we used both databases to calculate deaths relative to each other and performed final redactions and filtering. The result was four databases: DM-FS Officers and DM-FS civilians, as well as a cleaned version of them that is more conducive to quick, off-the-shelf analyses. These steps are discussed in more detail below.

**Table 1:** Table of Acronyms.

Acronym	Expansion
***Generic Acronyms***	
LEA	Law Enforcement Agency
FBI	Federal Bureau of Investigation
***Slain Officer Databases***	
CFOI	Census of Fatal and Occupational Injuries
LEOKA	Law Enforcement Officers Killed and Assaulted
FOS	Fallen Officer Search
***Slain Civilian Databases***	
NVSS	National Vital Statistics System
SHR	Supplemental Homicide Report
WP	Washington Post
MPV	Mapping Police Violence
FE	Fatal Encounters
***Other Databases***	
BJS	Bureau of Justice Statistics

First, we constructed a database that contained a list of law enforcement agencies (LEAs) in which an officer was slain by a civilian, which we termed DM-FS Officers. To construct this database, we (i) adjudicated between various slain officer databases, selecting the one that disclosed the most precise information concerning each individual officer’s death; (ii) extracted this data to a tabular format; and (iii) performed a technical validation exercise in which its anomalies and omissions were addressed. Second, we constructed a database of civilians that were fatally shot by an officer, which we termed DM-FS Civilians. To construct this database, we (i) adjudicated between fatal shooting databases and selected the three that did not contain the same serious underreporting concerns as the other two; (ii) merged the three databases together via an algorithmic-manual process; and (iii) performed a technical validation exercise in which discrepancies between the three were highlighted and corrected. We used three databases, rather than just one, as a means of correcting for the potential biases contained within each.

Third, we augmented both DM-FS Officers and DM-FS Civilians databases with additional data and operations. Namely, we used a BJS Crosswalk file and Python code to algorithmically link each LEA to a unique ID known as an originating agency identifier (ORI)^37^, in addition to importing other LEA-relevant information. We then calculated officer and civilian deaths relative to each other. Namely, we calculated the number of civilians that officers fatally shot both before and after an officer’s death (DM-FS Officers), and then we calculated the number of officers that civilians fatally shot both before and after a civilian’s death (DM-FS Civilians); this involved both databases, linking agencies between databases using ORI codes. Redactions were applied to conform with licensing, and after redactions, DM-FS was complete; the full DM-FS Officers and DM-FS Civilians were exported.

However, in its current state, DM-FS was designed to maximize optionality over ease-of-use. In other words, DM-FS was designed to contain too much data to give researchers the ability to filter it; it was not designed for quick, off-the-shelf analyses in which these filtering decisions were already made for them. To remedy this concern, we applied filtering to create a cleaned version of DM-FS–one that is less customizable, but one that is more conducive for quick analyses. Finally, these cleaned databases were exported into the same repository as the original ones, culminating in four final databases: the full version of DM-FS Officers and DM-FS Civilians, as well as the cleaned DM-FS Officers and DM-FS Civilians.

Each step is described in detail below, with details of the technical validation exercises appearing in the relevant section.

### DM-FS officers creation

The purpose of this subsection was to create a database of slain officers, which we termed DM-FS Officers. To assemble this database, we first wrestled with three existing databases on slain officers and chose the one that disclosed the most precise information concerning each officer’s death:the FBI’s Law Enforcement Officers Killed and Assaulted (“LEOKA”) reports^2–6^. Second, we downloaded LEOKA reports and extracted their content, and third, we addressed the anomalies and omissions contained therein via a technical validation exercise discussed in the corresponding section. This resulted in 232 LEAs that lost an officer between January 1, 2015, and December 31, 2020.

#### Database adjudication

DM-FS requires a list of LEAs in which an officer was slain, as well as the exact date of death. The exact date of death is exceptionally important because it will later be used to standardize fatal shootings between LEAs; for this reason, the database must be precise. There are three major databases that each disclose information about slain officers: the Census of Fatal Occupational Injuries (“CFOI”), the Fallen Officer Search (“FOS”), and Law Enforcement Officers Killed and Assaulted (“LEOKA”) Reports^38^. These three databases are maintained by the U.S. Bureau of Labor Statistics^26^, a private foundation called the National Law Enforcement Officers Memorial Fund^28^, and the Federal Bureau of Investigation (“FBI”)^23^ respectively.

A full review and adjudication of these three databases appears in the suppl. background materials; a summary of that review appears in Table 2. In short, we select the FBI’s LEOKA reports because it is the only database that provides access to rich, officer-level information. In other words, LEOKA is unique in that it posts a detailed vignette describing the final moments of each slain officer’s life^23^. These vignettes can be used to extract the exact date and time of an officer’s death, and they can also be used to verify that they were truly slain by a civilian. This is in contrast to CFOI, which only discloses aggregated statistics^26,38^, or FOS, which suffers from a serious problem of missing information (see suppl. background materials). LEOKA is not without fault; it may suffer from inaccuracies and omissions^39^, yet these are addressed via our technical validation.

**Table 2:** Summaries of Officers Slain Databases.

Name	Owner (Sector)	Summary	Key Issue(s)
Census of Fatal Occupational Injuries (CFOI)	US Bureau of Labor Statistics (Public)	Intended to be a national surveillance system of all US workplace-related deaths.	Deaths are aggregated. Cannot get civilian details behind each officer’s death.
Law Enforcement Officers Killed and Assaulted (LEOKA)*	Federal Bureau of Investigation (Public)	LEOKA is part of the FBI’s Uniform Crime Report, and it contains a detailed vignette of almost every officer who was intentionally slain in the line of duty.	May contain omissions and anomalies. Both were addressed in the technical validation section
Fallen Officer Search (FOS)	National Law Enforcement Officers Memorial Fund (Private)	A searchable database maintained by a private, right-leaning US foundation whose primary focus is to memorialize officers and attract new donors.	Overly inclusive of officers and deaths (e.g., military police, COVID deaths), and missing information prevents us from discarding this information.

Asterisk (*) indicates that a particular database was used to construct the initial list of slain officers.

#### Data download & extraction

After selecting LEOKA as the primary database of slain officers, its vignettes were extracted and copied into an Excel sheet for the inclusive, six-year period spanning from 2015 through 2020 by consulting the corresponding LEOKA reports^2–5,39,40^. We could not download LEOKA reports after 2020 because, from 2021 onward, the FBI anonymized LEOKA vignettes such that they no longer disclose the name of the agency that lost a slain officer^24^, thereby rendering them unusable for DM-FS.

To provide specific details on how we download LEOKA, we downloaded LEOKA vignettes from the FBI’s LEOKA repository (https://ucr.fbi.gov/leoka), in which we clicked on the corresponding year and then on the “Summaries of Officers Killed” hyperlink. For LEOKA reports published in 2020 onward, the FBI moved these to a new repository called the “Crime Explorer” web application (https://cde.ucr.cjis.gov/LATEST/webapp/). The web application is far more involved than the previous LEOKA repository. Thus, to download the 2020 LEOKA report, we undertook the following operation:

1. We visited the URL to open the Crime Explorer web application.
2. We hovered over “Data Explorer” on the top-right corner of the screen and clicked on “Law Enforcement Officers Killed and Assaulted” on the drop-down menu that appeared. This opened a new screen.
3. We clicked on the “LEOKA Annual Report Files for Download” hyperlink on that new screen, which opened yet another new screen.
4. Under the blue “Law Enforcement Officers Killed and Assaulted Annual Reports” heading, there was a series of drop-down menus in which we (i) selected the year 2020, (ii) clicked on “Officers Feloniously Killed”, and then (iii) clicked the “Download” button to download a zip file.
5. Finally, the zip file contained 54 files. We selected the file named “2020 LEOKA Officers Feloniously Killed Narratives_Final.docx,” which contained vignettes that were structured in the same way as those of previous years^2–5,39^.

Once these vignettes were downloaded, no filtering criteria were used. From every vignette, we extracted the following crucial details and pasted them in an Excel file: the LEA that employed the slain officer, the precise date of the officer’s death, details concerning the slain officer and civilian such as age, officer rank, and outcome of the fatal shooting, and the exact means by which an officer was slain, among others. This resulted in 232 LEAs that reported a slain officer.

### DM-FS civilians creation

The purpose of this subsection was to create a database of fatally shot civilians, which we termed DM-FS Civilians. To assemble this database, we first adjudicated between five pre-existing databases of civilians slain by officers. Of these five databases, we used the three crowdsourced databases because they do not contain the same serious underreporting concerns as the other two^16^. We selected three – rather than just one – so that we could use the contents of one database to “fact-check” the contents of the other two and vice-versa. Second, we merged the three databases together via an algorithmic-manual process, and third, we corrected any discrepancies arising between the databases so that they did not appear in DM-FS Civilians. Full details of these discrepancies and their corrections appear in the technical validation.

#### Database adjudication

A full review of existing databases of fatally shot civilians appears in the suppl. background materials. In short, there are five relevant databases: two are governmental, and three are crowdsourced in the sense that they are maintained by either citizen-volunteers or the news media. A summary of each appears in Table 3. We selected the three crowdsourced databases because they do not contain the same serious underreporting concerns as their governmental counterparts^16^, and they are also used most wildly in the literature (see suppl. background materials). These crowdsourced databases are Fatal Encounters (FE), Mapping Police Violence (MPV), and Washington Post (WP).

**Table 3:** Summaries of Major Fatal Shooting Databases.

Name	Owner (Sector)	Summary
National Vital Statistics System (NVSS)	U.S. Center for Disease Control (Public)	Government database maintained by the Center for Disease Control. Primary function is to record number of births and deaths in the US.
Supplemental Homicide Report (SHR)	Federal Bureau of Investigation (Public)	Part of FBI’s Uniform Crime Reports. Allows LEAs to voluntarily disclose civilians they fatally shot via justifiable homicides.
Washington Post (WP)*	Washington Post (Private)	US Newspaper that maintains database on all fatal shootings since 2015.
Mapping Police Violence (MPV)*	We the Protestors (Private)	Database maintained by left-leaning nonprofit organization. Contains a database that records all officer-involved homicides, excluding suicides.
Fatal Encounters (FE)*	Mr. D. Brian Burghart (Private)	Database records all officer-involved homicides, including suicides

Asterisk (*) indicates that a particular database was used to generate or verify the number of civilians that each LEA fatally shot.

Unfortunately, these crowdsourced databases contain distinct methodologies that govern which fatal shootings get reported, as summarized in Table 4, and others may be hosted by organizations that could have a political agenda^29,30^. It is unclear to what extent these concerns affect reportage, a concern compounded by a lack of comprehensive validation attempts. Thus, we elect to use all three databases so that the content of one can “fact-check” the content of the others. A full review of these crowdsourced databases, as well as a review of past validation attempts and further justification for combining the databases, appears in the suppl. background materials.

**Table 4:** Summary of the Three Major Crowdsourced Databases on Fatal Shootings.

	Washington Post (WP)	Mapping Police Violence (MPV)	Fatal Encounters (FE)
Types of officer-involved homicide included in database^55^	Fatal shootings only	Intentional and unintentional homicides	Any death during interaction with law enforcement
Date range	2015 to Present	2013 to Present	2000 to 12/31/2021
Data sources	Local news reports, LEA websites and social media, monitoring independent databases, & own investigations	Google alerts, which is then fed into a multi-tier filtering process	Data obtained via paid researchers, public access requests, and crowdsourcing
Discloses LEA responsible for civilian’s death?	Yes	Yes	Yes
Discloses victim’s name?	Yes	Yes	Yes
Discloses date of death?	Yes	Yes	Yes
Includes shootings by off-duty officers^51^?	No	Yes	Yes
Discloses source of fatal shooting?	No	Yes	Yes

#### Database download & algorithmic-manual merge

To download the three crowdsourced databases, we undertook the following procedure. For Fatal Encounters (FE)^22^, we went to their homepage (https://fatalencounters.org/) and clicked on the large blue icon directly above the “Download FE Database” URL. This opened a Google sheet, which was downloaded as an Excel file. For Mapping Police Violence (MPV)^25^, we went to their homepage (https://mappingpoliceviolence.org/) and clicked the “View the Data” URL. This opened an Airtable, which, upon clicking the three dots near the top of the screen, could be downloaded as a CSV file. For Washington Post (WP)^1^, we went to their GitHub site (https://github.com/washingtonpost/data-police-shootings/tree/master), opened up the “v2” folder, and downloaded both the “fatal-police-shootings-agencies.csv” and “fatal-police-shootings-data.csv” files. Unlike the other two databases, WP records fatal shootings using two CSV files: they record the LEA responsible for the fatal shooting as an ID number, and then they have a separate CSV file that maps the ID number to the LEA’s actual name. Thus, we merged the two CSV files into one by replacing the semi-arbitrary ID with the LEA’s actual name. The full details of which appear in our Python code.

As Table 4 illustrates, the crowdsourced databases sometimes record all manner of death in which law enforcement slays a civilian (i.e., FE, MPV), of which fatal shootings are a subset, whereas other databases just record fatal shootings (i.e., WP). Thus, we restricted all databases to just show civilians that were fatally shot via non-suicidal, which encompass the overwhelming majority of officer-involved homicides^16^. We achieved this through a series of filters. First, we filtered the three databases so that they only included deaths from January 1, 2015, to December 31, 2020. We did not go earlier than January 1, 2015, because that is when WP started recording fatal shootings^1^, and we did not go later than December 31, 2020, because from 2021 onward, the FBI started anonymizing LEOKA vignettes which rendered them unusable for DM-FS^41^. Second, we filtered MPV so that the “cause_of_death” column must have been set to “Gunshot”, thereby forcing MPV to only show fatal shootings. Third, we filtered FE to only include non-suicidal fatal shootings by (i) forcing the “Highest Level of Force” column to be set to “Gunshot” and (ii) requiring the “Intended Use of Force” column to not equal “Suicide”. Full details of this filtering appear in our Python code. Nevertheless, our filtering criteria resulted in the databases displaying roughly the same manner of death in which officer(s) slew a civilian: non-suicidal fatal shootings.

Finally, we merged the contents of the three databases in a way that validates their content. In other words, if a fatally shot civilian is shared between databases, then we could use the databases as a means of ‘fact-checking’ against each other–we could highlight, for example, if the databases disagree on information like date of death and age, and if so, we could investigate this value so that it is corrected in DM-FS. Unfortunately, therein lay a major issue: the databases *occasionally* disagreed on crucial identifying information like location, date of death, and even how a fatally shot civilian’s name was spelled. Thus, it was very difficult to link a fatally shot civilian across the databases in which he/she appeared.

To overcome this link issue, we crafted an extensive Python algorithm that linked individuals across databases so that their details could be merged and, when certain issues arose, we manually reviewed the link to cancel or allow the merger. Namely, our Python algorithm merged the databases state-by-state. Within a particular state, it then used conventional natural-language processing (NLP) techniques (e.g.^42^) – such as removing stop words, converting names to one case, and removing extra spaces – as well as approximate string matching in cases where exact string matching produced no results^43,44^ - to link civilian names across different databases. For example, if FE, MPV, and WP recorded that the civilian “John Doe”, “Jon Doe”, and “Jonny Doe” was fatally shot in Oregon, our algorithm would be able to identify this fatal shooting as belonging to the same civilian despite these minor perturbances in his name. When this sort of link arose, we manually reviewed whether the link was genuine and, in cases where it was not, we manually overrode the algorithm to prevent a merge from occurring; full details of this procedure, in addition to similar checks, appear in the technical validation.

Nevertheless, once a civilian was linked across databases, we used the databases as a means of “fact-checking” against each other. Namely, the algorithm checked whether crucial information about each civilian was equivalent in all the databases in which he/she appeared. For example, if a civilian appeared in FE and MPV, the algorithm checked whether the location of death and civilian age, among other variables, appeared the same in both databases. If so, the data was merged, but if not, it was flagged as a discrepancy that underwent manual review (see technical validation). In short, this merger resulted in 7,105 unique, fatally shot civilians from January 1, 2015, until December 31, 2020, with full results appearing in the technical validation.

### Augmenting DM-FS with additional data & operations

The purpose of this subsection was to augment both DM-FS Officers and Civilians with additional data and calculations before exporting them. First, we used a BJS crosswalk file to add an Originating Agency Identifier (“ORI”) code to each agency to allow us to link agencies together across databases and time, in addition to importing other information^37^. Second, we applied a few final steps to both DM-FS Officers and Civilians: we (i) calculated deaths 365 days before/after an officer death (DM-FS Officers) and civilian deaths (DM-FS Civilians), (ii) redacted the databases to comply with licensing, and (iii) filtered the databases such that there is a version of them that is more suitable for off-the-shelf analysis. The result of this subsection is four databases: the full version of DM-FS Officers and Civilians, as well as a cleaned version of DM-FS Officers and Civilians.

#### ORI Assignment

There are over 18,000 law enforcement agencies in America, and many agencies have overlapping names, functions, and jurisdiction^45,46^. To disentangle these overlapping agencies from each other, we obtained Originating Agency Identifier (“ORI”) codes for each agency. In other words, the FBI assigns a unique alphanumerical identifier to LEAs in America, and this identifier is known as an ORI code^47,48^. The ORI code is linked to a classification scheme that can help disentangle American law enforcement agencies from each other. Moreover, it can be used to link an agency to various government surveys like the 2008 Census of State and Local Law Enforcement Agencies (“CSLLEA”)^46^ and the Census. Thus, the ORI code serves as both a unique identifier and conduit by which an LEA can be linked to multiple other government databases. For these reasons, it is essential to assign these codes accurately.

To assign ORI codes, we downloaded a crosswalk file authored by the Bureau of Justice Statistics and hosted by the ICPSR (10.3886/ICPSR35158.V2)^37^. Namely, we visited the URL, clicked the “Download” button, and selected “delimited” as our chosen file format. This produced a zip file that, when extracted, contained a “35158-0001-Data.tsv” file. We used Excel to convert this file to a CSV.

Once the crosswalk file was downloaded and converted, we devised an algorithm that linked the LEAs in both DM-FS Civilians and Officers to the equivalent name in the crosswalk file^37^. Once a link was established, the ORI code was imported, in addition to the classification of that LEA derived thereof and other LEA-specific information. To establish this link, our algorithm filtered LEAs by state, and then it linked LEA names together using conventional NLP techniques (e.g.^42^) – such as removing stop words, converting names to one case, and removing extra spaces – as well as approximate string matching in cases where exact string matching produced no results^43,44^. Much like our database merge algorithm, we manually reviewed any link attempts that were the result of approximate string matching or if multiple link candidates were returned; and, in cases where the link was not valid, we manually overrode the algorithm to prevent an import from occurring (see technical validation). Using this automated procedure, we managed to assign the overwhelming majority of agencies an ORI. However, for the few agencies that were not assigned an ORI, we performed this ORI assignment manually and imported the relevant data. Full details appear in the technical validation.

#### Final steps

##### Standardized death count calculations

For DM-FS Officers, we calculated two crucial measurements. First, we calculated the number of civilians that the slain officer’s LEA fatally shot both 365 days before and after the officer’s death, as this is our primary measurement of how an officer’s death affects the number of civilians his/her colleagues fatally shot in response to death. Second, we calculated the number of officers that were slain both 365 days before and after each officer’s death *from that officer’s LEA* because, if the officer belongs to an LEA in which officers are regularly killed, his/her colleagues may respond to a death differently than an LEA in which an officer death is relatively novel. We undertook similar calculations for DM-FS Civilians, albeit these deaths were standardized relative to each civilian’s death rather than an officer’s. We used both databases to perform these calculations in which we linked agencies between databases using ORI codes. For example, to calculate the number of civilians that a slain officer’s LEA fatally shot in DM-FS Officers, we had to draw from our fatally shot civilian database, DM-FS Civilians, and vice-versa. Thus, the construction of both databases was essential to this calculation.

It is crucial to note a difference in death measurement between DM-FS Officers and DM-FS Civilians, as well as how we addressed it. Namely, DM-FS Officers was derived from LEOKA; it, therefore, contains all manner of death in which an officer was slain by a civilian. In contrast, DM-FS Civilians merely contains one manner of death in which civilians were slain by officers: fatal shootings. When calculating officer and civilian deaths, this creates a problem in which death count isn’t similarly measured between officers and civilians. Namely, civilians in DM-FS can only be killed via fatal shootings, whereas officers in DM-FS can be killed by all manner of death albeit most officers were slain via fatal shootings (215 officer deaths were fatal shootings out of 232 total, 92.67%). To overcome this measurement issue, we calculated two types of deaths whenever an officer-death-related measurement was used: (i) we calculated officer deaths *only due to fatal shootings* and (ii) all forms of officer death. Thus, these two officer-death-related measurements allow researchers to compare deaths between officers and civilians in a way in which the same type of death is measured, via fatal shootings, as well as in a way where officer death is defined more broadly.

Furthermore, all measurements are based on one-year measurement windows, 365 days, which was chosen because fatal shootings are rare events^7^, and a one-year measurement window is both common in the literature^16^ and should sufficiently measure these rare events. Moreover, these measurements exclude civilians who were fatally shot on the same day as the officer (day zero) in the case of DM-FS Officers, or officers who were fatally shot on the same day as a civilian in the case of DM-FS Civilians. This is due to the observation that, if a civilian and officer perished on the same day, this was almost certainly due to a civilian-initiated gunfight with the officer, thus resulting in multiple deaths–an observation confirmed by both the literature overall^49^ and individual LOEKA reports^2–6^. A simultaneous officer-civilian death is not an ideal measurement for fatal shootings *before* death; nor is it a good measurement for fatal shootings *after* death because people often do not learn of someone’s death until several hours or even days later^49^. For these reasons, day zero deaths were discarded.

##### Redactions

After we undertook these calculations, we applied a series of redactions to conform with licensing. As of the time of publication, we are negotiating licensing with ICPSR, the repository that hosts the BJS crosswalk file^37^. Until this license is finalized, we are unable to redistribute its data, which includes ORI codes, FIPS codes, and other demographic-related information concerning each LEA. Thus, we have removed all crosswalk data from our database and have replaced essential information with two derivatives. First, we replaced the ORI code with an anonymous identifier, and second, we replaced the law enforcement classification scheme contained therein with our own in-house classification scheme. DM-FS will be updated with the original ORIs, classification scheme, and related data via the Harvard Dataverse repository if a license has been obtained. In addition to these licensing-related redactions, we also removed the full name of each fatally shot civilian from DM-FS Civilians.

##### Exporting DM-FS & applying filtering to create cleaned DM-FS

After applying these redactions, DM-FS was complete. Namely, DM-FS Officers contains all officers that were slain by a civilian that appeared in LEOKA, whereas DM-FS Civilians contains all civilians that were fatally shot by an officer that appeared in at least one crowdsourced database (i.e., MPV, FE, WP). Moreover, all civilians and officers perished between January 1, 2015, and December 31, 2020. These two completed databases, therefore, were exported to the Harvard Dataverse.

However, DM-FS involves a design trade-off: it was designed to maximize optionality at the expense of ease-of-use. Namely, it was designed to include as much data as possible so that the end user – the researcher –has the ability to apply any filtering criteria that are suitable for his/her project. For example, in DM-FS Officers, we merely flagged LEAs whose deaths we recommend be excluded and explained our reason why; we did not actually delete them because we want to give researchers the ability to make that decision for themselves, thus, researchers will need to review the ‘exclude_death’ and ‘exclusion_reason’ columns. Moreover, DM-FS also lists deaths that cannot be effectively used to study death-modulated fatal shootings due to the time horizon. Specifically, a 365-day measurement window was used to count officer/civilian deaths, and the earliest datapoint was on January 1, 2015. Any data that occurred before January 1, 2016, therefore, would not have a sufficient 365-day measurement window; therefore, these measurements were given a -1 value, of which there are many of them. Thus, in its full form, DM-FS contains many inelegances that may need to undergo additional filtering and exclusions before it is effectively used in research. These inelegances allow researchers to customize it; yet, they make quick, off-the-shelf research difficult.

To offer the other end of this trade-off, we also launched DM-FS (cleaned). Namely, DM-FS (cleaned) is a derivative of DM-FS that is more user-friendly; it is DM-FS that has undergone additional filtering and exclusions. There is significantly less data in DM-FS (cleaned), hence, it is much harder to customize for research. However, the benefit of this filtering is that the data that is present is cleaner and easier to work with than DM-FS, thereby offering the other end of this design trade-off. Both DM-FS Officers and DM-FS Civilians underwent this cleaning process, hence, there are four databases total: DM-FS Officers and DM-FS Civilians, as well as DM-FS Officers (cleaned) and DM-FS Cleaned (cleaned).

To create DM-FS (cleaned), we applied two preprocessing techniques to both DM-FS Officers and DM-FS Civilians: we applied recommended exclusions, and then we removed non-essential columns. First, we applied our recommended exclusions to DM-FS Officers. Namely, we removed slain officers (i) whose date of death differed from their date of injury by more than six months (*n* = 7) (see technical validation), (ii) who belonged to an agency with an invalid ORI code, such as a federal agency or one in Puerto Rico (*n* = 16), and (iii) if the death occurred before January 1, 2016 (n = 29) or after December 31, 2019 (*n* = 21). These dates ensure a valid 365-day measurement window for counting deaths relative to the slain officer. We applied similar exclusions to DM-FS Civilians. Namely, we removed fatally shot civilians who (i) were fatally shot exclusively by agencies with an invalid ORI code (*n* = 189), such as federal agencies, and (ii) were fatally shot before January 1, 2016 (*n* = 1080) or after December 31, 2019 (*n* = 1,245) This resulted in a final database of 158 slain officers within DM-FS Officers (cleaned), 4,591 fatally shot civilians in DM-FS Civilians (cleaned), who perished between January 1, 2016 and December 31, 2019.

Second, we removed non-essential columns. Namely, within DM-FS Officers (cleaned), we removed 3 columns: we removed one column that indicated whether we recommend excluding the officer’s death, another column that disclosed the reason for our recommendation, and a final column that listed the anomaly, as all three columns were corrected. Second, within DM-FS Civilians (cleaned), we dropped 22 non-essential columns. 18 of those columns flagged which database was at fault for a discrepancy, 3 contained non-essential URLs, and the remaining column listed the magnitude of the date discrepancy if one arose between the databases. This resulted in a final database that contains 30 columns for DM-FS Officers (cleaned) and 64 columns for DM-FS Civilians (cleaned). Both cleaned databases were exported and uploaded to the Harvard Dataverse repository.

## Data Records

The entirety of DM-FS has been uploaded to the Harvard Dataverse (10.7910/DVN/7HK7HH)^50^. When accessing the URL, it is highly recommended to click the “Tree” button under the “File” heading to make the files more intelligible. Namely, there are three folders in the Dataverse: one for DM-FS Officers, another for DM-FS Civilians, and a final folder for oversized tables that support our technical validation exercises. The DM-FS Officers folder contains (i) the full version of DM-FS Officers, (ii) the cleaned version, and (iii) a codebook that describes both, whereas a similar logic applies to the DM-FS Civilians folder. DM-FS Officers and DM-FS Civilians are stored as Microsoft Excel (.xlsx) files in which its variables (columns) were grouped into categories for ease of use, with a list of those categories appearing in Table 5. In contrast, the technical validation folder contains five tables that are stored as “.tab” files, meaning they can be downloaded as a CSV, imported into R and other programming languages, as well as viewed on a web browser. Details of each folder appear below.

**Table 5:** Categories of Variables (Columns) within DM-FS, Separated by DM-FS Officers and DM-FS Civilians.

Category Name	Description	Example Variable(s)
***DM-FS: Officers***		
Slain Officer Details	Details concerning the slain officer	death_date, dead_cop_rank, dead_cop_experience_years
Slain Officer Counts: Past & Future	Number of officers in that LEA that were slain by civilians both before and after officer death	cops_agency_lost_365_days_before_death_fatal_shootings_only
Killer Details	Details pertaining to civilian that slew the officer/officers.	killers_weapon, killers_fate, killers_crim_history
Civilians Shot Before & After Officer Death	Number of civilians that officers in that LEA fatally shot both before and after officer death	civilians_fatally_shot_by_agency_365_days_after_death
LEA Details	Details of LEA that the slain officer(s) belonged to	LEA_name, LEA_state, LEA_region, LEA_type
Other	Other details concerning the incident	unique_vignette, notes, LEOKA_URL
***DM-FS: Civilians***		
Fatally Shot Civilian Details	Details concerning the fatally shot civilian	death_date, victim_age, victim_race, city_of_death
LEA Responsible #1-#5	Details concerning the LEA that initiated the fatal shooting. If multiple LEAs are responsible for the same fatal shooting, LEA Responsible will increment.	cops_from_agency_responsible_1_that_civillians_killed_365_days_before_death_for_fatal_shootings_only
Database Inclusion	Which crowdsourced database(s) the fatally shot civilian appeared in	included_in_fe, included_in_mpv
Databases at Fault for a Discrepancy	Which database(s) were at fault for a discrepancy	date_fe, date_mpv, date_wp
URLs	URLs to any source material	source_FE, source_MPV

A full list of variables that appear in each column appears in the Harvard Dataverse repository under the corresponding codebooks^50^.

### DM-FS officers

DM-FS Officers enable the exploration of how an officer’s death affects the number of civilians other officers fatally shoot each year, and under which circumstances. Each observation is an LEA that lost at least one officer, with corresponding details of the death – such as officer rank, manner of death, and the fate of the civilian that slew the officer - being extracted from LEOKA (see methods). In addition to DM-FS Officers, we have also launched DM-FS Officers (cleaned), which is a derivative of DM-FS that has undergone additional filtering to make it more suitable for quick, off-the-shelf analyses (see methods). Namely, DM-FS Officers (cleaned) is the same as DM-FS officers, except recommended exclusions were applied, non-essential columns were dropped, and the date range was more restricted to accommodate the 365-day measurement window. Finally, we have uploaded a codebook that describes each variable in DM-FS Officers. Details of all three are summarized below.

- **DM-FS Officers.xlsx**. Contains all agencies in which an officer was slain by a civilian between January 1, 2015, and December 31, 2020, as reported by LEOKA. This resulted in 232 LEAs (rows) with 33 variables (columns).
- **DM-FS Officers (cleaned).xlsx**. Contains all agencies in which an officer was slain by a civilian between January 1, 2016, and December 31, 2019, as reported by LEOKA. This further excludes instances of officers who belonged to an agency with an invalid ORI code – a federal agency or one in Puerto Rico – or those whose date of death was over six months from the date they sustained their injury. This results in 158 LEAs (rows) with 30 variables (columns).
- **DMFS - Officers Codebook.pdf**. After describing both datasets, the codebook contains an enumerated list of each variable (column) that appears in both DM-FS Officers and DM-FS Officers (cleaned), grouped by category. Namely, it explains the variable, discloses its type, and provides examples if they facilitate understanding.

### DM-FS civilians

DM-FS Civilians enables the exploration of how a civilian’s death affects the number of officers that other civilians kill each year, and under which circumstances. Namely, each observation is an individual civilian who was fatally shot by an LEA, with details of the civilian and LEA derived from the three crowdsourced databases (i.e., FE, MPV, and WP) (see methods and technical validation). Similar to DM-FS Officers, we also launched a clean version of DM-FS civilians, which is a derivative of DM-FS civilians in which additional filtering was applied and non-essential columns were dropped to make it more conducive to quick, off-the-shelf analysis. Finally, we have uploaded a codebook that describes each variable within both DM-FS Civilian databases. All three files are summarized below.

- **DM-FS Civilians.xlsx**. Contains all civilians that were fatally shot by an officer between January 1, 2015, and December 31, 2020, as reported by the three crowdsourced databases (FE, MPV, and WP) that were filtered to display fatal shootings that were non-suicides. This resulted in 7,105 fatally shot civilians (rows) with 86 variables (columns).
- **DM-FS Civilians (Cleaned).xlsx**. Contains all civilians that were fatally shot by an officer between January 1, 2016, and December 31, 2019, as reported by the three crowdsourced databases (FE, MPV, and WP) that were filtered to display non-suicidal fatal shootings. This further excludes fatally shot civilians in which all agencies responsible had invalid ORI codes, such as fatal shootings committed solely by federal agencies. This resulted in 4,591 fatally shot civilians (rows) with 64 variables (columns).
- **DMFS - Civilians Codebook.pdf**. After describing both datasets, the codebook contains an enumerated list of each variable (column) that appears in both DM-FS Civilians and DM-FS Civilians (cleaned). Namely, it explains the variable, discloses its type, and provides examples, if applicable.

### Technical validation tables

Finally, the repository contains technical validation tables that can be downloaded as a CSV file and/or viewed via a web browser^50^. These tables collectively provide evidence that our database assembly procedure was successful, and they are referenced and explained in the corresponding portions of the technical validation section. An enumerated list of these files appears below.

- **Repository Table 1** **- LEOKA Anomalies and Their Correction.tab**. This table contains a list of anomalies that we identified and manually corrected within the FBI’s LEOKA reports so that they did not re-appear in DM-FS Officers. These mainly consisted of minor adjustments to the officer’s date of death (see technical validation).
- **Repository Table 2A** **- Database Merge - Results of Approximate String Matching for Civilian Names that Failed Exact Matching.tab**. To construct DM-FS Civilians, we merged the three crowdsourced databases together (i.e., FE, MPV, WP), and we employed two validation procedures to ensure the efficacy of the database merge. This table displays the full results of our first validation procedure.
- **Repository Table 2B** **- Database Merge - Results of Approximate String Matching for Civilian Names that Failed Exact Matching.tab**. This table displays the full results of the second validation procedure that was used to ensure the efficacy of the database merge used to construct DM-FS Civilians.
- **Repository Table 3A** **- ORI Assignment - Linkage Review.tab**. We created an algorithm that linked LEAs from DM-FS Officers and DM-FS Civilians to their corresponding entry in the BJS crosswalk file^37^ so their ORIs – and other relevant information – can be imported. The Linkage Review was our means of ensuring that our algorithm functioned successfully, with its full results appearing in that table.
- **Repository Table 3B** **- ORI Assignment – Manual Assignment.tab**. This table lists all LEAs that our algorithm was unable to assign a valid ORI code from both DM-FS Officers and Civilians. We attempted to assign each agency that appeared in this table an ORI code by manually searching the BJS crosswalk file, and if an ORI code was assigned, its assignment and rationale were noted.
- **Repository Table Descriptions.txt**. This file contains a detailed description for each of the repository tables.

## Technical Validation

DM-FS was created through three major subsections, and each subsection involved at least one technical validation exercise. First, we validated DM-FS Officers by screening LEOKA for anomalies and omissions, which, upon their identification, were noted and addressed. Second, we validated DM-FS Civilians using validation exercises that occurred (i) during the database merge and (ii) after the merge. Third, we validated the database augmentation exercise in which LEAs were assigned an ORI by (i) manually reviewing certain links and (ii) manually assigning ORI links in cases where the algorithm returned no match. These validation exercises help ensure that every major portion of DM-FS is technically sound.

Finally, it is crucial to note that both DM-FS Officers and Civilians were exported as both full versions and cleaned versions; however, this export did not occur *until the very end of the process*. Thus, all technical validation exercises that occurred on DM-FS Officers also occurred for DM-FS Officers (cleaned), and the same holds true for DM-FS Civilians and DM-FS Civilians (cleaned).

### DM-FS officers creation

DM-FS Officers contained a list of LEAs in which an officer was slain between January 1, 2015, and December 31, 2020, as well as a detailed vignette describing the death of an officer. This list underwent a two-step validation exercise: we first manually scrutinized each vignette for anomalies, and second, we searched for omissions. Results and their corrections appear below.

#### LEOKA anomalies

We manually read each LEOKA vignette at least three times per vignette and noted all anomalies. Of the 232 LEAs that experienced an officer death, 35 contained anomalies (15.09% of LEAs). Namely, one LEA displayed an agency name that did not appear anywhere online; this name was corrected by consulting online documents. Another LEA reported the death of an officer who perished while off-duty; this was the only LEA in LEOKA that reported the death of an off-duty officer during our measurement period, hence it was flagged. We further flagged three LEAs as having a “killer” anomaly in which two civilians attempted to kill an officer rather than just one. This was incompatible with how DM-FS Officers was structured, as it only allows for the entry of one killer. Thus, for these three LEAs, we truncated it such that the most likely killer appeared in DM-FS rather than both killers. This correction may be contested; however, it only applies to the overwhelming minority (1.29%) of all LEAs that lost an officer, and we flagged it such these agencies can easily be filtered out.

Finally, the overwhelming majority of these anomalies were date-related (85.71%, 30 anomalies out of 35 total), in which the date an officer was slain was different than the date he/she received his/her fatal injuries. Most (*n* = 22) date anomalies were corrected by using the date that the officer was mortally wounded rather than his/her death date because in these cases, the death date occurred less than two weeks after the officer was wounded. However, some date anomalies (*n* = 8) were far greater: an officer from the Chicago Police Department, for example, died 33 years after receiving his initial injuries. When these major discrepancies occurred, the officer’s death date was changed to reflect the date he/she sustained the injury, and it was flagged for exclusion. An enumerated list of LEOKA anomalies and their correction appears in Repository Table 1 - LEOKA Anomalies and Their Correction.tab^50^.

#### LEOKA omissions

In addition to anomalies, we screened LEOKA for omissions. In other words, the FBI may not include a slain officer’s vignette in its LEOKA reports due to court gag orders or privacy concerns^39^. This vignette omission is crucial because vignettes represent the only means of extracting individual-level data on officer death: without vignettes, we could not extract the LEA that employed the slain officer, nor could we learn the officer’s death date and means of death. Thus, an omitted vignette would render an officer’s death unusable, and unfortunately, this concern materialized in the present study. Indeed, LEOKA withheld the vignettes of 49 slain officers during this six-year period: 4 in 2015, 10 in 2016, 2 in 2017, 5 in 2018, 7 in 2019, and 21 in 2020^2–5,39,40^. It is unclear how many LEAs these slain officers belong to because multiple officers would often die in the same instance (e.g.^3^); these 49 omitted officer deaths, therefore, likely translate to less than 49 omitted LEAs. Assuming the worst-case scenario – that each officer represents a unique LEA – then these omissions suggest that 16.84% of all LEAs that had a slain officer were omitted: 49 omissions out of 291 total. This relatively small omission percentage should not seriously bias results, especially given that this small percentage represents a worst-case scenario; the actual percentage may be much smaller. Due to both the difficulty of correcting for these omissions and the relatively nominal nature of the problem, these omissions were left acknowledged but uncorrected.

### DM-FS civilians creation

DM-FS Civilians was created by merging the three existing crowdsourced databases, with any discrepancies noted and corrected. Our validation exercise was two-fold. First, we undertook two validation exercises during the database merge to ensure its efficacy. Second, we gathered statistics after the database merge to further validate its efficacy, noting and correcting any discrepancies that arose during the merge. Details of both appear below.

#### Database download & algorithmic-manual merge

Our algorithm linked civilians together between databases using (i) state of death and (ii) name. More precise identifiers like date or city of death were difficult to use because the databases occasionally disagreed on this crucial identifying information. Once a civilian was linked, his/her data was merged, and any discrepancies between databases were noted. The success of the database merge, therefore, is fundamentally determined by civilians being successfully linked across the databases in which they appear. Thus, we erected safeguards to ensure that each link was genuine: (i) we manually reviewed the results of all approximate string matches and (ii) we manually reviewed suspicious links. In each case where the link was not valid, we would override the algorithm such that a database merge would not occur.

##### Manual review of approximate string matches

First, our algorithm attempted to link a civilian across the databases in which he/she appeared based on his/her *exact name* in the first instance. If there were no links, then our algorithm used approximate string matching to link this civilian across databases despite minor perturbances in his/her name^43,44^. However, approximate string matching can sometimes introduce false positives–of extremely similar names being incorrectly linked. Thus, each time an approximate string match triggered a link, we manually verified that the link was genuine.

Namely, our algorithm attempted to make 13,454 links. First, a civilian from FE would attempt to be linked to his/her equivalent in MPV and WP, thereby triggering two link attempts. There were 6,590 civilians in FE, hence, 13,180 link attempts were triggered (6,590 * 2). Second, all *unlinked* civilians from MPV would attempt to be linked to his/her equivalent in WP. There were 274 unlinked civilians in MPV, hence, 274 link attempts were triggered, bringing the total to 13,454 link attempts.

Only 291 of these 13,454 link attempts (2.16%) used approximate string matching to salvage a link when exact string matching failed. Upon manual review, we determined that 287 (98.63%) of these 291 link attempts were genuine: the same civilian was indeed listed in multiple databases, albeit minor spelling differences prevented him/her from being linked using exact string matching. To determine whether the name truly belonged to the same civilian, we restricted the approximate string matching to only search individuals who were fatally shot in the same state; thus, “John Doe” could only be matched with “Johnny Doe” if they were fatally shot in the same state. This provided additional confidence that the approximate string match was genuine; however, we also consulted news stories and used additional information like dates of death and city of death to verify the link was genuine. For the four cases in which a link was not genuine, we manually overrode the algorithm to prevent a merge from occurring. Full results of our approximate string matching decisions appear in Repository Table 2A - Database Merge - Results of Approximate String Matching for Civilian Names that Failed Exact Matching.tab^50^.

##### Manual review of suspicious links

In addition to manually reviewing the approximate string matches, we manually reviewed links that we labeled as suspicious. To be labeled as suspicious, a link must have either (i) returned multiple results from the same database or (ii) the date of death must have differed by more than three days between the link candidates. This procedure occurred *after* any approximate string matching was applied; thus, if we somehow linked multiple civilians with the same name from one database to a single civilian from another, it would be flagged as suspicious. If either condition was triggered, we used additional information like date of death and city to determine if a link was genuine.

The overwhelming majority of links did *not* trigger this manual review procedure. Of the 13,454 link attempts, only 130 of these attempts (0.97%) were flagged as suspicious and underwent manual review. The overwhelming majority of these suspicious link attempts were because the civilian’s name was not disclosed by police; thus, there were multiple civilians named “Not Disclosed” who were fatally shot in the same state, requiring us to manually review these links. For this reason, the overwhelming majority of these suspicious links were not valid (86.92%, 113 link attempts out of 130 total), in which we once again overrode the algorithm to prevent a merge from occurring. The full results of our manual review of suspicious links procedure appear in Repository Table 2B - Database Merge - Results of Approximate String Matching for Civilian Names that Failed Exact Matching.tab^50^.

#### Post-merge validation

The manual review of suspicious links, as well as the manual review of approximate string matches, helped combat against names being incorrectly linked. To evaluate whether our database merge successfully linked individuals, we gathered statistics that measured the overall efficacy of our merger. We also gathered statistics on any discrepancies that our algorithm flagged between the three databases, as well as corrected those discrepancies.

##### Overall merge statistics

To validate the efficacy of our database merge algorithm, we measured the percentage of fatally shot civilians from one database who were successfully linked to civilians in another. Namely, 93.46% of civilians listed in Fatal Encounters were successfully linked to the same civilian in Mapping Police Violence and/or Washington Post (6,159 linked out of 6,590 total); whereas the equivalent link rate for Mapping Police Violence was 97.53% (5,913 linked out of 6,063 total) and Washington Post was 96.05% (5,711 linked out of 5,946 total). This high link rate means that, for the overwhelming majority of fatally shot civilians in FE, there was exactly one other civilian in MPV and/or WP who (i) shared the same name as them, (ii) was fatally shot in the same state, and (iii) were fatally shot within three days of each other, lest a manual review get triggered; the same logic holds for MPV and WP. The relative rigidity of this selection criteria can suggest that each link was genuine; and the fact that our algorithm linked so many civilians together between databases may be indicative of its success.

**Fig. 3:**
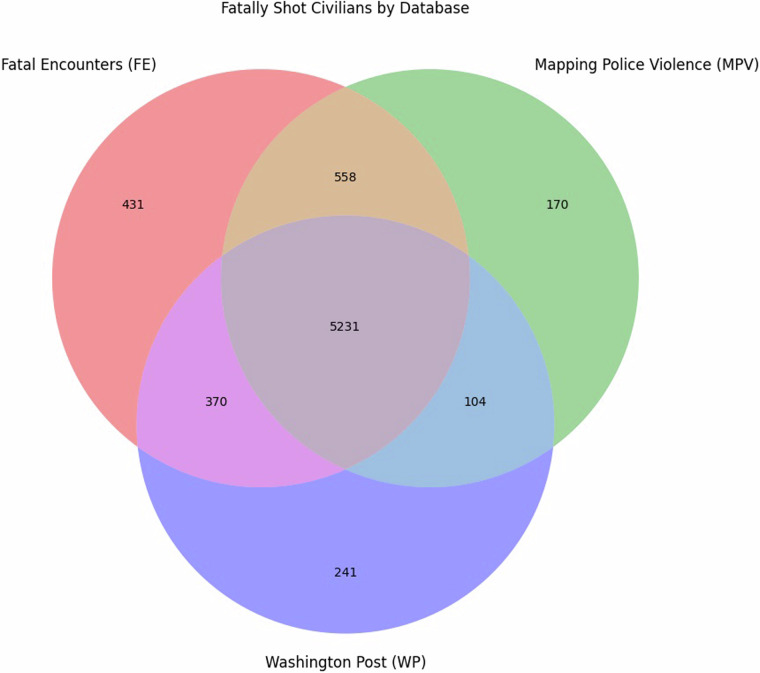
Venn Diagram Depicting Number of Fatally Shot Civilians by Database. Numbers in the Venn Diagram indicate the amount of fatally shot civilians that appear in all three crowdsourced databases (i.e., FE, MPV, WP), appear exclusively in two databases, or appear exclusively in one.

While merging the databases, our algorithm flagged which database(s) each fatally shot civilian appeared in. This flagging procedure revealed that there were many civilians who were exclusive to one or two databases, absent from the third–an observation further explored in Fig. 3.

Figure 3 illustrates that most of the 7,105 fatally shot civilians appeared in all three databases (*n* = 5231, 73.62%). However, over a quarter of fatally shot civilians were *not* in all three databases, with 6.07% appearing exclusively in Fatal Encounters (*n* = 431), 2.34% appearing exclusively in Mapping Police Violence (*n* = 170), and 4.61% in Washington Post (*n* = 241). Taken together, these statistics suggest that 11.85% of fatally shot civilians (*n* = 842) are exclusive to one database, whereas the remaining 14.52% (*n* = 1032) only appear in two of the three databases. Given that fatal shooting research sometimes relies perfunctorily on one database alone (e.g.^17–19^), this finding could suggest that these studies may be somewhat confounded, as researchers who uncritically choose one database over the other(s) may have missed a non-negligible portion of fatal shootings.

The finding that 26.2% (*n* = 1,842) of fatally shot civilians did *not* appear in three databases is surprising because our filtering criteria should have restricted the databases to only displaying roughly equivalent datapoints–fatally shot civilians that were non-suicides. The reason why these fatally shot civilians did not appear in the three databases is unclear. It could be, for example, that different databases have different inclusion criteria – such as FE and MPV tracking off-duty officers whereas WP does not^51^–and there was no elegant means of filtering out these different inclusion criteria when merging the databases. It could also be the result of genuine omissions, where the methodology of one database may capture a fatally shot civilian that the other two do not capture. This latter possibility seems likely, especially since WP and MPV have their own idiosyncratic emails that civilians can use to independently report a fatally shot civilian that may otherwise go undetected^1,25,52^, thereby resulting in a database-exclusive fatal shooting. To uncover why these datapoints do not appear in all three databases, a research team would likely need to manually investigate these 1,842 fatally shot civilians, a worthwhile task beyond the scope of the present descriptor.

Crucially, however, DM-FS Civilians corrects this concern because it derives its data from all three databases. Moreover, as discussed in the Usage Notes, it contains a filter that allows researchers to only access fatally shot civilians that appear in one database, in two of the three databases, or in all three databases jointly. In this sense, DM-FS allows researchers to choose which methodology they prefer, allowing them to select fatally shot civilians recorded from a methodology that meets the criteria of their research project.

##### Identify & correct discrepancies

In addition to highlighting database-exclusive fatal shootings, our algorithm also highlighted discrepancies between the databases. Namely, for each fatally shot civilian, the algorithm checked to ensure that the civilian’s date of death, gender, age, race, city of death, and agency/agencies responsible for that death were the same between databases. If there was a discrepancy that could not be corrected, it was flagged for manual review. To correct these flagged discrepancies, we consult all databases in which a civilian appeared, in addition to outside news articles to learn the source of the discrepancy. For each discrepancy, we noted which database was at fault. Thus, our 7,105 fatally shot civilians produced a total of 3,944 discrepancies, which are enumerated in Table 6.

**Table 6:** Databases at Fault for a Discrepancy by Type.

Discrepancy Type	Database at Fault for Discrepancy			Total Discrepancy Faults (Between Databases)
	*Fatal Encounters (FE)*	*Mapping Police Violence (MPV)*	*Washington Post (WP)*	
*Date*	134	36	232	402
*City*	117	250	907	1,274
*Gender*	7	3	8	18
*Age*	115	127	214	456
*Race*	366	187	276	829
*Agency Responsible*	164	231	570	965
Total Discrepancy Faults (Within One Database)	903	834	2,207	3,944

Discrepancies were identified via the full DM-FS Civilian database, which contains 7,105 civilians who were fatally shot between January 1, 2015, and December 31, 2020, inclusive. Discrepancy count indicates the number of instances in which a database was at fault for a discrepancy. Total discrepancy faults are not mutually exclusive both between and within databases. For example, a fatally shot civilian can have his gender correctly identified by FE but not WP or MPV, hence, one row can lead to two discrepancy faults. Moreover, one fatally shot civilian can lead to multiple discrepancies within one database, such as a database listing the age, gender, and race incorrectly.

MPV appears to have been at fault for the least discrepancies, with WP being at fault for the most. Most of WP’s discrepancies were location-based. Many of these location-based discrepancies arose because WP used the county rather than the city of death, thus producing a discrepancy. When removing location-based discrepancies, the difference in discrepancies becomes less extreme; however, WP is still at fault for far more discrepancies than the other two databases. WP may be at fault for an especially high number of discrepancies because it does not disclose the source(s) behind a fatal shooting. In other words, when a discrepancy arose between WP and at least one other database, we often sided with FE and/or MPV; we often labeled WP as being at fault because WP did not disclose its source(s), hence, we could not locate where it got its information and thereby side with it. Therefore, it is possible that WP may have had fewer discrepancies if it had listed the sources it used to gather details behind a fatally shot civilian.

Nevertheless, the crucial implication is that these discrepancies were identified and corrected for DM-FS; they are no longer material to research that draws from our database. Unfortunately, some fatal shooting scholars draw from one of these crowdsourced databases alone (e.g.^17–19^), suggesting that these past research attempts could be confounded by these uncorrected discrepancies, in addition to the problem of database-exclusive fatal shootings. This assertion is highly speculative because it is unclear to what extent these discrepancies affect past research results. However, the sheer number of discrepancies in Table 6 highlights the crucial importance of validating a crowdsourced database, as well as potentially using multiple databases to validate findings.

### Augmenting DM-FS with additional data & operations

After both DM-FS Officers and Civilians were validated, we assigned each LEA an ORI code before performing any additional calculations or operations. ORI codes were essential to DM-FS; thus, technical validation was performed to ensure its efficacy.

#### ORI assignment

The ORI code was a crucial identifier that allowed us to link LEAs across different databases and times. To accurately assign each LEA an ORI code, we crafted a Python algorithm that linked each LEA that appeared in DM-FS Officers and DM-FS Civilians with its counterpart in the Bureau of Justice Statistics (BJS) crosswalk file^37^. Namely, the algorithm filtered each database by state. It then used conventional NLP processing techniques^42^ and exact string matching to link an LEA from DM-FS Officers or Civilians to its equivalent entry in the crosswalk file. Approximate string matching was used in cases where exact string matching produced no links^43,44^. Once a link was established, the ORI code and other relevant variables were imported into DM-FS. Much like the database merge, the accuracy of these links is crucial because an incorrect link would create an incorrect import in which an erroneous ORI and associated information would appear in DM-FS. Therefore, to ensure each link was genuine, we undertook two procedures: (i) we manually reviewed certain link attempts, overriding the algorithm in cases of an incorrect link to prevent an erroneous, and (ii) we manually assigned ORIs to LEAs in which the algorithm could not create a link. Details of both appear below.

##### Manual link review

To avoid false links, we manually reviewed each link when one of two conditions arose. First, any time an approximate string match produced a link, the link was manually verified: we inspected both agency names and ORI codes to confirm whether they were, in fact, the same agency. To reach this point, the agencies being linked must reside in the same state, thereby providing additional confidence that this link is, in fact, genuine. Second, if there were multiple link candidates– if there were multiple agencies in the crosswalk file that had the same name and resided in the same state as an LEA in DM-FS – then we manually reviewed each candidate to assess which link was genuine (if any were genuine at all).

There were 7,920 link attempts. Namely, there were 232 LEAs in DM-FS Officers. There were also 7,688 LEAs in DM-FS Civilians: while there were only 7,105 fatally shot civilians, there were sometimes *multiple* LEAs responsible for a single fatal shooting, thereby resulting in 7,688 responsible LEAs. 10.63% of these linkage attempts (842 out of 7,920 total) resulted in a link review. We manually verified that 31.59% of links were genuine (266 genuine attempts out of 842 total attempts) and overrode the algorithm for the remaining 68.41% to prevent an erroneous import (576 incorrect attempts out of 842 total attempts). Most of these false links were driven by a small handful of LEAs undergoing an approximate string match and thereby resulting in a serious number of similar-sounding LEA link candidates that needed to be rejected. For example, the LEA “California State University” did not have an exact ORI match; thus, our algorithm listed 12 different branches of California State University Police (e.g., Sacramento, Fullerton, Los Angeles, etc.), and we had to reject all links that did not map onto the location revealed in the news article, thereby resulting in one approximate string match producing *many* false links. Thus, through this procedure, we assigned an ORI to 7,508 LEAs: 90.95% of LEAs in DM-FS Officers were assigned an ORI (211 out of 232 total LEAs), whereas the equivalent rate for DM-FS Civilians is 94.91% (7,297 out of 7,688 total LEAs). Full results of our manual link procedure appear in Repository Table 3A - ORI Assignment - Linkage Review.tab^50^.

##### Manual assignment

However, there were still 412 LEAs that were not assigned a crucial ORI code: 391 in DM-FS Civilians, and 21 in DM-FS Officers. To assign these agencies an ORI, we manually searched the crosswalk file for the LEA’s name, and then we manually inserted the ORI code, and related variables were variables into DM-FS. This resulted in assigning an ORI code to 23.81% of LEAs missing an ORI code in DM-FS Officers (5 out of 21 total) and 38.11% of LEAs missing an ORI code in DM-FS Civilians (149 out of 391 total). Each time an ORI code was assigned, we made a note as to the rationale. We could not assign an ORI code to the remaining LEAs because, in the overwhelming majority of cases, the LEA was a federal agency – such as the FBI, US Marshals, or US Border Patrol – and federal agencies were not assigned an ORI code in the BJS crosswalk file^37^. Most of the remaining agencies without an ORI code were in Puerto Rico where they were also not assigned an ORI code; or they were somewhat niche, unconventional LEAs that, for whatever reason, failed to appear in the crosswalk, such as the Court Officers of New York State.

Thus, after manual assignment, 96.74% (7,662 out of 7,920 total) LEAs received an ORI code: 93.10% in DM-FS Officers (216 out of 232 total) and 96.85% in DM-FS Civilians (7446 out of 7688 total). This very high match rate – in tandem with (i) manual link review and (ii) manual assignment – provides confidence that our ORI assignment procedure was successful. Full details of each manual assignment and lack thereof appear in Repository Table 3B - ORI Assignment – Manual Assignment.tab^50^.

## Usage Notes

A few caveats are in order before DM-FS is used in research. First, DM-FS is intended for fatal encounter and fatal shooting research. It is not intended for gun violence research because gun violence research requires data on both fatal and non-fatal shootings. DM-FS only contains data on fatal shootings, and fatal shootings may not accurately represent non-fatal shootings^53,54^ because non-random factors like gun caliber, officer training, and willingness to provide medical attention all affect whether a non-fatal shooting becomes fatal^7,16^. Thus, fatal shootings and non-fatal shootings can be viewed as relatively distinct phenomena, and without data on the latter, it is difficult to use DM-FS to contribute to gun violence scholarship overall.

That said, DM-FS can still contribute to the scholarship on fatal shootings. A police department that does not provide immediate medical attention to a civilian they shot^7^, for example, would likely experience far more fatal shootings than non-fatal shootings, making that agency far more prevalent in DM-FS and thereby likely to be detected by researchers. Indeed, the scholastic field of US fatal shootings has recently undergone a great awakening of scholastic and public interest^7^, suggesting there is much interest in this field, and research on fatal shootings can still save lives, even if it may not generalize to gun violence research overall. Thus, DM-FS may still make an important contribution, albeit one difficult to generalize to the gun violence scholarship overall.

Second, researchers should consult the codebooks on the Harvard Dataverse repository before using both DM-FS Officers and Civilians^50^. The codebook is especially important for DM-FS Officers because it contains caveats about using certain variables. In other words, the tabular data in DM-FS Officers were extracted from the unstructured text within LEOKA vignettes. When reading these vignettes, we noticed peculiarities with some of the variables that appear in DM-FS Officers. For example, it seemed somewhat arbitrary as to whether an LEA labeled a fatal shooting as an ambush, and the vignettes do not appear to give a comprehensive account of the civilian’s criminal history; both observations affect how the ‘killer_initiated_ambush’ and the ‘killer_crim_history’ variables are interpreted, and both are noted in the codebook. Indeed, the codebook contains an enumerated list of variables used as well as caveats in interpreting all of those we identified as caveat-worthy, making this codebook an essential read before DM-FS-driven fatal shooting research is undertaken.

Third, when using DM-FS Civilians, researchers should think carefully about which type of fatal shootings they would like to measure and adjust DM-FS Civilians accordingly. By default, DM-FS Civilians records civilians that were fatally shot by police that were non-suicides, as they are recorded by the three crowdsourced databases. However, all three databases use different methodologies to amass fatal shootings, as well as slightly different inclusion criteria (Table 4); this has resulted in them recording different instances of fatally shot civilians, as shown in Fig. 3. Moreover, American law enforcement is diverse^45^, and DM-FS Civilians records all types of LEAs that fatally shot a civilian. This includes state agencies, county sheriff’s offices, local police departments, and even hyper-local agencies like those patrolling a K-12 school district. Not all of these departments may be of interest to researchers.

To wrestle with these different methodologies and LEAs, DM-FS Civilians contains a filter feature that allows researchers to filter the database by methodology and LEA type. Regarding the latter, researchers can filter DM-FS so that it only displays civilians who were fatally shot by a certain LEA type or combinations of LEA types. To use this filter feature, researchers simply need to use the “filter” feature in Excel to force the columns “agency_responsible_X_LEA_TYPE” and “agency_responsible_X_LEA_SUBTYPE” to equal the LEA type that they would like to research, where ‘X’ is a placeholder for the agency responsible for the fatal shooting, such as agency_responsible_1, agency_responsible_2, etc. The same procedure can be implemented in computer code, and an equivalent procedure also exists for DM-FS Officers.

A similar logic holds for filtering DM-FS Civilians by database methodology. Namely, researchers can filter DM-FS so that it only displays fatally shot civilians that are present in one database, in two databases, or in all three databases together. To use this filter feature, researchers simply need to use the “filter” feature in Excel to force the columns “included_in_fe”, “included_in_mpv”, and “included_in_wp” to equal True or False, with the same procedure once again being implementable in computer code. Thus, this filter can be used to obtain a list of fatally shot civilians that were exclusive to a particular database’s methodology, thereby allowing for a more precise definition of fatal shootings.

The filter feature may also enable interesting comparisons between databases. For instance, researchers could use DM-FS to study fatally shot civilians using only those that appear in FE. Researchers could analyze those findings, and then they could conduct *the same analysis* using fatally shot civilians that appeared in MPV and/or WP to see if those findings hold. It would be fascinating, for example, to see if these inter-database findings diverge, and if so, this would provide some early evidence that studies that have exclusively drawn from one of these databases could potentially be confounded (e.g.^17–19^). Regardless, DM-FS can serve as the foundation for future fatal shooting studies that validate their findings using all three crowdsourced databases rather than just one, potentially leading to more robust findings for the scholastic field.

Finally, the filter feature suggests that DM-FS can also be used to study differences between the three crowdsourced databases with respect to which type of fatal shooting they recorded. This line of inquiry is important, given that the databases contain a non-negligible portion of fatal shootings that appear exclusively on one or two databases but not the other(s) (Fig. 3). If this inquiry is pursued, one should keep in mind that all three crowdsourced databases are valuable. In other words, FE and MPV both have an extensive, thoughtful, and transparent methodology on how they record fatally shot civilians, and they provide sufficient transparency such that their dataset can be easily double-checked^22,52^. By comparison, WP is far less transparent – both with respect to its relatively sparse methodology and with respect to failing to disclose its sources – however, it adds value in that it can conduct its own investigations on fatally shot civilians^1^. Thus, future fatal shooting studies should consider viewing these three databases as different instruments that complement each other, which can collectively be used to gain a more holistic measurement of fatally shot civilians.

## Supplementary information

Supplemental Information

## Data Availability

All code was written in Python 3.10. Full code and replication advice appear on GitHub (https://github.com/jjv31/dmfs/tree/main).
