# Supplementary material for: DM-FS: A Comprehensive Database on Death-Modulated Fatal Shootings
Source: Sci Data. 2025 Mar 3;12:370. doi: 10.1038/s41597-025-04649-x (PMC11876296; doi:10.1038/s41597-025-04649-x)
Supplement: Supplementary file 1 — Supplemental Information [file 41597_2025_4649_MOESM1_ESM.pdf]

SUPPLEMENTAL MATERIALS

DM-FS: A Comprehensive Database on Death-Modulated Fatal Shootings

Jacob Verrey[1*]

[1] Cantab. Institute of Criminology, University of Cambridge, Sidgwick Ave, Cambridge CB3 9DA, +44 1223 335360; jjv31@cam.ac.uk

* Corresponding author. *Email:* jjv31@cam.ac.uk (J. Verrey).

Contents

Supplemental Background Materials 1
Review of Slain Officer Databases. 1
Review of Slain Civilian Databases. 2
Review of Past Validation Attempts of Crowdsourced Databases. 6
Supplemental References 8

Supplemental Background Materials

Review of Slain Officer Databases

As discussed in the main text, there are three major databases that each disclose information about slain officers: the Census of Fatal Occupational Injuries ("CFOI"), the Fallen Officer Search ("FOS"), and Law Enforcement Officers Killed and Assaulted ("LEOKA") reports (Tiesman et al., 2013). These three databases are maintained by the U.S. Bureau of Labor Statistics (U.S. Bureau of Labor Statistics, 2016), the National Law Enforcement Officers Memorial Fund (National Law Enforcement Officers Memorial Fund, 2019), and the Federal Bureau of Investigation (Federal Bureau of Investigation, 2004) respectively.

We first consider the Census of Fatal Occupational Injuries ("CFOI"), which is intended to be a national surveillance system of all US workplace-related deaths. Namely, it aggregates workplace deaths based on industry, occupation, and unique injury codes [1,2]. Unfortunately, our database demands individual datapoints that reveal each officer's death date and LEA affiliation, as well as additional data to verify the authenticity of each story. Because individual-level data cannot be disentangled from CFOI's aggregated statistics, this database was discarded.

The Fallen Officer Search ("FOS") is a searchable database of officer deaths managed by the National Law Enforcement Officers Memorial Fund [3], a right-leaning private foundation. Some of the key motivations behind the database are to memorialize law enforcement and attract new donors [3]. For this reason and others, it appears to be overly inclusive of its contents. For example, the database does not confine itself to traditional law enforcement: it includes atypical officers like game-wardens and military [1], which seldom engage in fatally shooting civilians. Moreover, it includes atypical officer deaths, such as officers succumbing to COVID [4]. Database filters and web scraping can be used to discard irrelevant information, yet this database also suffers from a problem

of missing information --- LEA affiliation and exact cause of death are missing for a surprising number of officers. Missing information, therefore, prohibits us from filtering the irrelevant data that exists on this database, meaning it cannot be used.

The final database was the Law Enforcement Officers Killed and Assaulted ("LEOKA") Reports. Since the 1960s, LEOKA has been part of the Federal Bureau of Investigation's ("FBI") Uniform Crime Report --- the primary means of gathering national crime statistics in the United States that involves self-reporting crime to the FBI [5,6]. In addition to crime, agencies can also self-report data on slain officers, which subsequently becomes part of LEOKA. LEOKA is unique in the sense that it discloses a surprising amount of detail about each officer's death. Specifically, the FBI posts a detailed vignette of each officer's death in its death [7]. These vignettes disclose the story that portrays the end of an officer's life; it allows one to extract the exact time and date of each officer's death, as well as their means of death and the LEA that employed them. Indeed, the vignettes often portray the officer's final moments of life, allowing us to scrutinize each officer's death to verify whether they truly were slain by a civilian. Because these vignettes provide rich access to individual data, LEOKA was selected as the sole source of information concerning slain officers. LEOKA, however, occasionally suffers from omissions [8], yet these are addressed via the technical validation section.

Review of Slain Civilian Databases.

Official Databases. As discussed in the main text, five different databases contain information concerning slain officers: two are governmental, and three are crowdsourced. The two official national databases on civilians slain by US Law Enforcement are the National Vital Statistics System ("NVSS") and the Supplementary Homicide Report ("SHR"), which are managed by the Center for Disease Control and Federal Bureau of Investigation respectively [7,9]. The former is a

decentralized database whose primary function is to record the number of births and deaths in the United States [9,10], while the latter is part of the Uniform Crime Report, in which local law enforcement agencies voluntarily self-report data to the FBI.

NVSS may not be suitable for investigating fatal shootings. Specifically, the NVSS obtains information on officer deaths from death certificates [9,10], and death certificates are almost exclusively filled out by physicians [11,12]. Physicians may only have access to the final cause of death when completing the death certificate – they may know that a patient died due to a gunshot wound for example, but they may not know that wound came from an officer – and thus, the NVSS may underreport fatal shootings. Unfortunately, multiple recent empirical investigations confirmed this speculation: the NVSS captures less than half of all fatal shootings [11–13]. This underreporting is serious enough to discard NVSS for the purpose of building DM-FS.

The FBI's Supplementary Homicide Report ("SHR") can also be used to understand fatal shootings. The SHR lists fatal shootings as justifiable homicides, and it has historically served as the primary source of fatal shooting data among criminologists and practitioners [5,6,14]. It works via self-reportage: just as agencies self-report crime via the Uniform Crime Report, they can also self-report the number of civilians that their LEA fatally shot via the SHR [7]. Unfortunately, fatal shootings may be one of the most contentious and embarrassing issues facing law enforcement [15–17], and self-reporting contentious and embarrassing issues can facilitate underreporting. This speculation has an empirical reality: the SHR has been found to record less than half of all fatal shootings [14,18]. Moreover, this underreporting may be especially relevant in small towns [19], locations where most fatal shootings take place [20,21], suggesting that SHR also suffers from a selection bias. Because of both severe underreporting and this selection bias, SHR was discarded.

Crowdsourced Databases. Given the dearth of reliable official sources, fatal shooting researchers have turned to crowdsourced databases [14,22]. These databases are crowdsourced in the sense that they are maintained by either citizen-volunteers or news media; these databases are manually updated upon news of a fatal shooting as depicted in a news article, a court document, or a social media posting among other forms of dissemination [23–25]. These crowdsourced databases have been shown to report a far greater number of fatal shootings than official sources, and for this reason, they are used widely in the field [13,14,18,26–31]. The most widely used crowdsourced databases are the Washington Post, Fatal Encounters, and Mapping Police Violence [32]. We discuss each below, with a summary of these differences appearing in Table 4 in the main text.

The Washington Post ("WP") is an American newspaper that has been logging fatal police shootings from 2015 to the present [25]. It learns of fatal shootings via social media postings, news accounts, and police reports. Its database records the fatal shooting victim's name, the date of death, and basic demographic details. However, unlike the other two databases, it does not disclose the source(s) which it used to gather information about the fatally shot civilian. For this reason, it is the least transparent of the three major databases.

Mapping Police Violence ("MPV") is a database that records all forms of officer-involved death, not just fatal shootings. It is maintained by the left-leaning 501(c)(3) nonprofit organization called We the Protestors under the project name known as Campaign Zero [24,33]. We the Protestors is a left-of-center organization that is associated with the Black Lives Matter movement [34], and it is attempting to "build a world beyond policing" [33] --- as such, MPV may be influenced by this political agenda. Regardless, Mapping Police Violence records all officer-involved homicides from 2013 to the present.

Fatal Encounters ("FE") is a database originally maintained by journalist D. Brian Burghart and teams of individuals [23]. FE has been recording all forms of officer-involved homicides since 2000, and while it did not officially report a discontinuation date, its last entry was on 12/31/2021. Of the three databases, Fatal Encounters employs the most extensive methodology to learn of new fatal shootings. It first employs paid researchers to assemble an initial list of all officer-involved deaths – not just fatal shootings - from various open sources, and this initial list was then vetted by D. Brian Burghart. Second, it used a crowdsourcing platform in which the public can voluntarily report an officer-involved homicide to the team, in which all datapoints are once again verified by D. Brian Burghart. Third, it employed a separate team to send Freedom of Information Act requests to various LEAs to obtain additional information on all forms of officer-involved homicides --- information that may not have been picked up by the news media. Indeed, this methodology is so thorough that contemporary scholars have hailed it as "the largest collection of officer-involved homicides in the United States" [28].

Selecting All Three Crowdsourced Databases. In summary, WP, MPV, and FE are the most commonly used databases in fatal shooting scholarship [32], in part because they do not suffer from the same serious underreporting concerns as their governmental counterparts [14]. However, they each contain different mechanisms for recording fatal shootings (see Table 4) and are hosted by organizations that may be influenced by a political agendas [33]. It is unclear to what extent these methodological and political differences affect fatal shooting reportage --- a fear compounded by the lack of acceptable verification attempts on these three databases (see suppl. background for review). To remedy this lack of verification, we use all three databases to obtain and validate a list of civilians that each LEA fatally shot.

This combined approach does not guarantee that the final list of fatally shot civilians is free from existing biases. A reporting bias present in all three databases, for example, will not be assuaged via this procedure. However, these crowdsourced databases may be our best approximation of a national list of US fatal shooting victims [14,32], and thus, they will still be used. Moreover, the aggregation of thoughtful estimations tends to produce a better approximation of the truth than an individual estimation alone, as popularized via the wisdom of the crowds finding in psychology [35]. Thus, thoughtfully using these three databases should still correct for some methodological or political biases idiosyncratic to a particular database, and in this sense, our approach represents an improvement over using one database alone.

Review of Past Validation Attempts of Crowdsourced Databases

Washington Post ("WP"), Mapping Police Violence ("MPV"), and Fatal Encounters ("FE") are the three crowdsourced databases that list the civilians fatally shot by US law enforcement. They contain distinct methodologies (see Table 4 in the main text) and are the most commonly used databases in the fatal shooting scholarship [32]. Given their importance to the field, other researchers have attempted to validate them, yet none of these validation attempts are sufficiently comprehensive for the purpose of constructing DM-FS. We review the major attempts below.

Typically, past work has attempted to verify these databases by extracting a small, non-random sample of slain civilians and matching it to records in the corresponding city/cities [26,27,29]. Although these studies find no major discrepancies between a database and city records, their samples may not generalize to the LEA list from Phase One. First, they suffer from an issue of small sample size. Second, these authors typically rely on records from big cities, and many LEAs from Phase One are small towns. Big cities are systematically different from small towns in ways that could confound fatal shootings and their reportage [19,21,36], meaning the verification of big city records

may not be generalizable to those of small towns. Comer and Ingram have presented the most comprehensive verification effort of these databases to date, and they find that these databases contain roughly the same *number* of slain civilians [32]. The individual details of each case – such as the death date or who was killed – remain unverified, meaning even the most comprehensive verification attempt cannot be generalized to DM-FS.

Supplemental References

1. Tiesman, H. M., Swedler, D. I., Konda, S. & Pollack, K. M. Fatal occupational injuries among U.S. law enforcement officers: A comparison of national surveillance systems. *American J Industrial Med* **56**, 693–700 (2013).
2. U.S. Bureau of Labor Statistics. *Census of Fatal Occupational Injuries*. 37 https://www.bls.gov/opub/hom/cfoi/pdf/cfoi.pdf (2016).
3. National Law Enforcement Officers Memorial Fund. *NLEOMF Annual Report 2019*. https://nleomf.org/wp-content/uploads/2021/08/Annual-Report-2019_final.pdf (2019).
4. National Law Enforcement Officers Memorial Fund. *Law Enforcement Officer Deaths: Preliminary 2021*. https://nleomf.org/wpcontent/uploads/2022/01/2021-EOY-Fatality-Report-Final-web.pdf (2021).
5. Bell, M. C. Next-Generation Policing Research: Three Propositions. *Journal of Economic Perspectives* **35**, 29–48 (2021).
6. Maltz, M. D. *Bridging Gaps in Police Crime Data*. (DIANE Publishing, 2000).
7. Federal Bureau of Investigation. *Uniform Crime Reporting Handbook*. https://ucr.fbi.gov/additional-ucr-publications/ucr_handbook.pdf (2004).
8. Federal Bureau of Investigation. *Privacy Impact Assessment for the Law Enforcement Officers Killed and Assaulted (LEOKA) Program*. https://www.fbi.gov/file-repository/pia-leokaprogram.pdf (2019).
9. Ventura, S. J. The US national vital statistics system: transitioning into the 21st century, 1990–2017. (2018).
10. Center for Disease Control. National Vital Statistics System.

11. Global Burden of Disease 2019. Fatal police violence by race and state in the USA, 1980–2019: a network meta-regression. *The Lancet* **398**, 1239–1255 (2021).
12. Loftin, C., McDowall, D. & Xie, M. Underreporting of Homicides by Police in the United States, 1976-2013. *Homicide Studies* **21**, 159–174 (2017).
13. Feldman, J. M., Gruskin, S., Coull, B. A. & Krieger, N. Quantifying underreporting of law-enforcement-related deaths in United States vital statistics and news-media-based data sources: A capture–recapture analysis. *PLoS Med* **14**, e1002399 (2017).
14. Zimring, F. E. *When Police Kill*. (Harvard University Press, 2017).
15. Community Oriented Policing Services. *Emerging Use of Force Issues: Balancing Public and Officer Safety*. https://portal.cops.usdoj.gov/resourcecenter/RIC/Publications/cops-p232-pub.pdf (2012).
16. Klinger, D. *Into the Kill Zone: A Cop's Eye View of Deadly Force*. (John Wiley & Sons, 2012).
17. Oliver, W. M. *Policing America: An Introduction*. (Aspen Publishing, 2020).
18. Zimring, F. E. How many killings by police. *U. Chi. Legal F.* 691 (2016).
19. Williams, H. E., Bowman, S. W. & Jung, J. T. The Limitations of Government Databases for Analyzing Fatal Officer-Involved Shootings in the United States. *Criminal Justice Policy Review* **30**, 201–222 (2019).
20. Reaves, B. *Census of State and Local Law Enforcement Agencies, 2008*. https://bjs.ojp.gov/library/publications/census-state-and-local-law-enforcement-agencies-2008 (2011).
21. Sherman, L. Small is dangerous: community size and police shooting deaths. (2015).

22. Sherman, L. W. Reducing Fatal Police Shootings as System Crashes: Research, Theory, and Practice. *Annu. Rev. Criminol.* **1**, 421–449 (2018).
23. Burghart, B. Fatal Encounters: A step toward creating an impartial, comprehensive and searchable national database of people killed during interactions with police. Fatal Encounters (2019).
24. Campaign Zero. Mapping Police Violence. Mapping Police Violence (2022).
25. Tate, J. *et al.* Fatal Force Database. Washington Post (2022).
26. Baćak, V., Mausolf, J. G. & Schwarz, C. How Comprehensive Are Media-Based Data on Police Officer–Involved Shootings? *J Interpers Violence* **36**, NP10055–NP10065 (2021).
27. Feldman, J. M., Gruskin, S., Coull, B. A. & Krieger, N. Killed by Police: Validity of Media-Based Data and Misclassification of Death Certificates in Massachusetts, 2004–2016. *Am J Public Health* **107**, 1624–1626 (2017).
28. Finch, B. K. *et al.* "Using Crowd-Sourced Data to Explore Police-Related-Deaths in the United States (2000–2017): The Case of Fatal Encounters". *OHD* **6**, 1 (2019).
29. Ozkan, T., Worrall, J. L. & Zettler, H. Validating media-driven and crowdsourced police shooting data: a research note. *Journal of Crime and Justice* **41**, 334–345 (2018).
30. Renner, M. L. Using Multiple Flawed Measures to Construct Valid and Reliable Rates of Homicide by Police. *Homicide Studies* **23**, 20–40 (2019).
31. Tregle, B., Nix, J. & Alpert, G. P. Disparity does not mean bias: Making sense of observed racial disparities in fatal officer-involved shootings with multiple benchmarks. in *Contemporary Issues in American Policing* 18–31 (Routledge, 2021).

32. Comer, B. P. & Ingram, J. R. Comparing Fatal Encounters, Mapping Police Violence, and Washington Post Fatal Police Shooting Data from 2015–2019: A Research Note. *Criminal Justice Review* **48**, 249–261 (2023).
33. We the Protestors. Campaign Zero. *Campaign Zero* https://campaignzero.org/#vision.
34. Adele Peters. Meet The Startup Building The Digital Civil Rights Movement. *Fast ompany* (2016).
35. Surowiecki, J. The wisdom of crowds: Why the many are smarter than the few and how collective wisdom shapes business, economies, societies, and nations. *The wisdom of crowds: Why the many are smarter than the few and how collective wisdom shapes business, economies, societies, and nations.* xxi, 296–xxi, 296 (2004).
36. Glaeser, E. L. & Sacerdote, B. Why is there more crime in cities? *Journal of political economy* **107**, S225–S258 (1999).
